# Effects of metformin use on total mortality in patients with type 2 diabetes and chronic obstructive pulmonary disease: A matched-subject design

**DOI:** 10.1371/journal.pone.0204859

**Published:** 2018-10-04

**Authors:** Fu-Shun Yen, Weishan Chen, James Cheng-Chung Wei, Chih-Cheng Hsu, Chii-Min Hwu

**Affiliations:** 1 Dr. Yen’s Clinic, Taoyuan City, Taiwan; 2 Management Office for Health Data, China Medical University Hospital, Taichung, Taiwan; 3 College of Medicine, China Medical University, Taichung, Taiwan; 4 Division of Allergy, Immunology and Rheumatology, Chung Shan Medical University Hospital, Taichung, Taiwan; 5 Institute of Population Health Sciences, National Health Research Institutes, Zhunan, Miaoli, Taiwan; 6 Department of Health Services Administration, China Medical University, Taichung, Taiwan; 7 Department of Family Medicine, Min-Sheng General Hospital, Taoyuan, Taiwan; 8 Faculty of Medicine, National Yang-Ming University School of Medicine, Taipei, Taiwan; 9 Section of Endocrinology and Metabolism, Department of Medicine, Taipei Veterans General Hospital, Taipei, Taiwan; National and Kapodistrian University of Athens, GREECE

## Abstract

**Backgrounds:**

Few studies have investigated the therapeutic effects of metformin in patients with type 2 diabetes mellitus (T2DM) and chronic obstructive pulmonary disease (COPD). We compared the risk of all-cause mortality between metformin users and nonusers.

**Methods:**

We conducted a retrospective cohort study for patients with T2DM and COPD who were enrolled between January 1, 2000 and June 30, 2012. Individuals with exacerbated symptoms who were hospitalized or sent to the emergency department (ED) were identified as having exacerbated COPD; outpatient claims were identified as having stable COPD. A total of 40,597 metformin users and 39,529 nonusers comprised the cohort of stable COPD; 14,001 metformin users and 21,613 nonusers comprised the cohort of exacerbated COPD. Users and nonusers were matched using propensity score (1:1). Our primary outcome was all-cause mortality.

**Results:**

A total of 19,505 metformin users were matched to 19,505 nonusers in the cohort of diabetes with stable COPD. The mean follow-up time was 3.91 years. All-cause mortality was reported in 1326 and 1609 metformin users and nonusers, respectively. After multivariate adjustment, metformin users had lower risk of mortality (adjusted hazard ratio [aHR] = 0.84, *p* < 0.0001). Metformin users had significantly lower risk of noncardiovascular death (aHR = 0.86, *p* = 0.0008). A total of 7721 metformin users were matched to 7721 nonusers in the cohort of diabetes with exacerbated COPD. The mean follow-up time was 3.18 years. All-cause mortality was reported in 1567 and 1865 metformin users and nonusers, respectively. After multivariate adjustment, metformin users had significantly lower risk of mortality (aHR = 0.89, *p* = 0.002) and cardiovascular death (aHR = 0.70, *p* = 0.01).

**Conclusion:**

This large-series, nationwide cohort study demonstrated that metformin use could significantly lower the risk of all-cause mortality in patients with T2DM and either stable or exacerbated COPD.

## Introduction

Chronic obstructive pulmonary disease (COPD) is a progressive inflammatory lung disease that blocks airflow [1]. Approximately 8%–22% of adults aged 40 years and older have COPD [2], and the estimated global prevalence is 11.7%. Approximately 400 million people around the world are affected by COPD [3]. In 2015, COPD was the third leading cause of age-standardized mortality for both sexes, with approximately 3.2 million deaths caused by this disease [4]. Because of inflammatory processes and the use of high-dose corticosteroids, COPD might increase the risk of developing type 2 diabetes mellitus (T2DM) [5, 6]. Among patients with COPD, 1.6%–16% had diabetes, and the prevalence increased as lung function deteriorated [7].

The prevalence of diabetes mellitus (DM) is escalating. According to the International Diabetes Federation, currently 415 million adults have diabetes globally, and this number is expected increase to 642 million by 2040 [8]. Diabetes mellitus, as a chronic disease with many complications, is a heavy burden for both patients and societies around the world [9]. Approximately 10% of patients with diabetes have COPD [10]. T2DM could worsen the progression and prognosis of COPD through the direct effects of hyperglycemia on pulmonary function, inflammation, and susceptibility to bacterial infections [11]. Adequate control of blood glucose levels to reduce hyperglycemia would improve COPD prognosis.

Metformin lowers the blood glucose level by increasing insulin-stimulated glucose uptake in skeletal muscles and adipocytes and reducing hepatic glucose output by inhibiting gluconeogenesis and glycogenolysis [12]. In addition to improving glucose metabolism, metformin could inhibit inflammatory processes and airway inflammation by activating adenosine monophosphate–associated protein kinase (AMPK) [13]. Metformin could also increase antioxidant defense [14] and decrease oxidative stress [15]. An open-label metformin study in patients with COPD demonstrated that metformin could improve health status and respiratory muscle strength [16]. One retrospective study also suggested that metformin could improve forced vital capacity [17].

In the individualized treatment of T2DM, COPD is a crucial comorbidity, but there are few clinical studies specifically regarding patients with combined diabetes and COPD [18, 19]. Therefore, we conducted this nationwide cohort study to observe the safety and long-term outcomes of metformin use in patients with T2DM and COPD.

## Methods

### Study design and participants

This retrospective cohort study was conducted in Taiwan. The National Health Insurance Research Database (NHIRD) included health care data gathered from 99% of the Taiwanese population (approximately 23 million people) [20]. Encrypted information recorded in the NHIRD included residency area; date of birth; sex; diagnostic codes according to the International Classification of Diseases, Ninth Revision, Clinical Modification (ICD-9-CM); drug prescriptions; and medical procedures. In this study, we focused on diabetic patients selected from the Longitudinal Cohort of Diabetes Patients (LHDB). The LHDB is a part of the NHIRD. From this database, 120,000 patients with newly diagnosed diabetes were randomly selected each year from 1999 to 2012, and their medical records were collected from 1996 to 2013. This study was approved by the Institutional Review Board of China Medical University in central Taiwan (CMUH104-REC2-115). We were waived to receive informed consent from the participants because all information that might be used to identify patients or care providers was encrypted before release. Identifying any patients or care givers at any level from this dataset was not possible.

We included patients who had a record of T2DM and COPD in the LHDB between January 1, 2000 and December 31, 2012; the end of this study was patients’ withdrawal from the health insurance, occurrence of the outcome of interest, or until December 31, 2013. COPD (ICD-9-CM codes: 491, 492, and 496) was diagnosed on the basis of data regarding outpatient claims, emergency department (ED) visits, or inpatients claims. Patients with COPD and three or more outpatient diagnoses were classified as having stable COPD, whereas patients whose initial diagnosis was from at least one ED visit or hospitalization were classified as having exacerbated COPD [21]. We excluded patients who were younger than 40 years or older than 100 years, were withdrawn from the national health insurance (NHI), or had been diagnosed with metabolic acidosis (ICD-9-CM code: 276.2) before index date. We also excluded patients who had been diagnosed with DM or COPD before January 1, 2000 to accurately calculate DM and COPD duration.

### Procedures

We defined the date of concurrent diagnosis of DM and COPD as the new date. Patients who took metformin for at least 30 days after the new date were identified as metformin users. Patients who did not use any metformin were identified as metformin nonusers. The date of taking metformin after the new date was defined as the index date for study entry. We identified variables that might influence the risk of death: demographic characteristics; comorbidities diagnosed before the index date; and use of specific classes of drugs such as other oral antidiabetic drugs (OAD, including: sulfonylureas, meglitinides, α-glucosidase inhibitor, thiazolidinedione, dipeptidyl peptidase-4 inhibitors, and insulin), antihypertensive drugs, COPD drugs, statins, and aspirin. We used the Charlson comorbidity index (CCI); which is a weighted index that assesses the number and severity of myocardial, vascular, pulmonary, neurologic, endocrine, renal, liver, gastrointestinal, cancer, immune, coagulopathy, and rheumatologic diseases; to quantify patients’ comorbidity profiles [22]. The diabetes complications severity index (DCSI) scores the severity of diabetic complications including retinopathy; nephropathy; neuropathy; cerebrovascular, cardiovascular (CV), or peripheral vascular diseases; and ketoacidosis, hyperosmolar, or other coma [23]; the DCSI was used to evaluate the severity of diabetes. The CCI and DCSI were calculated according to the subjects’ status 1 year before the index date.

### Outcomes

For assessing the primary outcome of all-cause mortality, we used discharge diagnosis of the last hospitalization before death. Death was also considered if a hospitalized patient adopted an “against advice discharge” due to a critical illness. The date of discharge was defined as the date of mortality to estimate the mortality rates. We also checked the last primary diagnosis of discharge three months before death to search for the causes of death [24]. According to the Standardized Definitions for Endpoint Events in Cardiovascular Trials [25], the causes of CV death are as follows: 1. Death caused by ischemic heart disease (myocardial infarction [MI], ICD-9-CM codes: 410, 411.0, 412, and 429.79; coronary artery disease [CAD], ICD-9-CM codes: 410–414 and 429.2). 2. Sudden cardiac death (sudden cardiac arrest, ICD-9-CM code: V12.53; cardiac arrhythmia, ICD-9-CM code: 42**7)**. 3. Death caused by heart failure (HF, ICD-9-CM codes: 398.91, 402.01, 402.11, 402.91, and 428). 4. Death caused by stroke (ICD-9-CM codes: 430–438). 5. Death caused by CV procedure (ICD-9-CM codes: 668.1 and 997.1). 6. Death caused by CV hemorrhage (aortic aneurysm and dissection, ICD-9-CM code: 441; cardiac tamponade, ICD-9-CM code: 423.3). 7. Death by other CV causes (arterial embolism and thrombosis, ICD-9-CM code: 444). For non-CV causes of death, we assessed cancers (ICD-9-CM codes: 140–208), lung cancers (ICD-9-CM code: 162), respiratory disorders (ICD-9-CM codes: 518.81, 518.82, 518.85, 786.09, 799.1, 96.71, 96.72, 96.04, and 93.9), bacterial pneumonia (ICD-9-CM codes: 481, 486, 482.41, and 482.8), and others. Cases for which we could not obtain the last primary diagnosis for three months before death were classified with undetermined causes of death.

We considered metabolic acidosis, including lactic acidosis and other illness of metabolic acidosis, as at least one admission with this diagnosis (ICD-9-CM code: 276.2) to see the safety of metformin use in patients with T2DM and COPD.

### Statistical analyses

We used propensity score matching to balance the two groups with respect to known confounders to augment their comparability [26]. Some inevitable confounding factors might remain disproportionally in these study groups; but, propensity score matching could ideally balance the distributions of measured covariates as much as a randomized control trial [27]. We estimated the propensity score for every patient through nonparsimonious multivariable logistic regression, using receipt of metformin as the dependent variable. We incorporated 28 clinically relevant covariates into our analysis as independent variables (all baseline characteristics are presented in Tables 1 and 2). The nearest-neighbor algorithm was applied to construct matched pairs, assuming that the proportion of 0.995–1.0 was perfect [28].

**Table 1:** Demographic and baseline characteristics of the stable COPD cohort.

	Before propensity score match				*p* value	After propensity score match				*p* value
	Metformin users (n = 40597)		Metformin non-users (n = 39529)			Metformin users (n = 19505)		Metformin non-users (n = 19505)		
	n	%	n	%		n	%	n	%	
Gender					<0.0001					0.46
Female	18850	46.4	19269	48.7		9252	47.4	9325	47.8	
Male	21747	53.6	20260	51.3		10253	52.6	10180	52.2	
Age					<0.0001					0.0004
40–64	22003	54.2	16577	41.9		9066	46.5	9417	48.3	
≧65	18594	45.8	22952	58.1		10439	53.5	10088	51.7	
mean(SD)	63.2(10.8)		67.4(11.6)		<0.0001	65.4(10.7)		65.4(11.4)		0.57
Diabetes duration (years)										
Mean (SD)	7.01(3.61)		6.86(3.79)		<0.0001	6.40(3.74)		6.54(3.71)		0.0002
Charlson comorbidity index					<0.0001					0.76
0	24335	59.9	20365	51.5		11200	57.4	11157	57.2	
1	6184	15.2	5636	14.3		2797	14.3	2775	14.2	
≧2	10078	24.8	13528	34.2		5508	28.2	5573	28.6	
DCSI score					<0.0001					0.87
0	30989	76.3	31486	79.7		15154	77.7	15135	77.6	
1	4433	10.9	3425	8.66		1922	9.9	1953	10.0	
≧2	5175	12.7	4618	11.7		2429	12.5	2417	12.4	
Antihypertensive drugs										
ACEI/ARB	27026	66.6	16965	42.9	<0.0001	10892	55.8	10829	55.5	0.52
β-blockers	18791	46.3	13665	34.6	<0.0001	7898	40.5	8030	41.2	0.17
Calcium-channel blockers	24842	61.2	18116	45.8	<0.0001	10684	54.8	10719	55.0	0.72
Diuretics	13425	33.1	8414	21.3	<0.0001	5082	26.1	5153	26.4	0.41
Other antihypertensives	6689	16.5	4541	11.5	<0.0001	2591	13.3	2611	13.4	0.77
Antidiabetic drugs										
Oral antidiabetic agents					<0.0001					0.12
0–1	23845	58.7	38038	96.2		17943	92.0	18039	92.5	
2	9788	24.1	1178	2.98		1251	6.41	1153	5.91	
>2	6964	17.2	313	0.79		311	1.59	313	1.60	
Insulin	14121	34.8	4568	11.6	<0.0001	3564	18.3	3415	17.5	0.049
COPD drugs										
Short-actingβ_2_bronchodilators	1825	4.50	1168	2.95	<0.0001	709	3.63	712	3.65	0.94
Long-actingβ_2_bronchodilators	112	0.28	102	0.26	0.62	49	0.25	57	0.29	0.44
Anticholinergic agent	825	2.03	696	1.76	0.005	369	1.89	370	1.90	0.97
Inhaled corticosteroids	978	2.41	578	1.46	<0.0001	390	2.00	394	2.02	0.89
systemic corticosteroid	11442	28.2	7389	18.7	<0.0001	4405	22.6	4420	22.7	0.86
Methylxanthine	14206	35.0	8866	22.4	<0.0001	5376	27.6	5425	27.8	0.58
Other drugs										
Statin	23616	58.2	11270	28.5	<0.0001	8723	44.7	8839	45.3	0.24
Aspirin	18656	46.0	11585	29.3	<0.0001	7276	37.3	7329	37.6	0.58

Abbreviation: DCSI, diabetes complications severity index; ACEI, angiotensin converting enzyme–inhibitor; ARB, angiotensin II receptor–blocker.

**Table 2:** Demographic and baseline characteristics of the exacerbated COPD cohort.

	Before propensity score match				*p* value	After propensity score match				*p* value
	Metformin users (n = 14001)		Metformin non-users (n = 21613)			Metformin users (n = 7721)		Metformin non-users (n = 7721)		
	n	%	n	%		n	%	n	%	
Gender					0.97					0.32
Female	4686	33.5	7238	33.5		2562	33.2	2620	33.9	
Male	9315	66.5	14375	66.5		5159	66.8	5101	66.1	
Age					<0.0001					0.93
40–64	4797	34.3	3876	17.9		1976	25.6	1971	25.5	
≧65	9204	65.7	17737	82.1		5745	74.4	5750	74.5	
mean(SD)	68.9(11.6)		75.1(11.1)		<0.0001	71.8(11.0)		71.8(11.4)		0.96
Diabetes duration (years)										
Mean (SD)	6.20(3.64)		6.27(3.75)		0.06	5.67(3.73)		5.75(3.56)		0.15
Charlson comorbidity index					<0.0001					0.87
0	1346	9.61	1248	5.77		585	7.58	579	7.50	
1	2499	17.8	3117	14.4		1240	16.1	1219	15.8	
≧2	10156	72.5	17248	79.8		5896	76.4	5923	76.7	
DCSI score					<0.0001					0.80
0	9049	64.6	15207	70.4		5117	66.3	5133	66.5	
1	1456	10.4	1710	7.91		736	9.53	712	9.22	
≧2	3496	25.0	4696	21.7		1868	24.2	1876	24.3	
Antihypertensive drugs										
ACEI/ARB	8985	64.2	8610	39.8	<0.0001	4164	53.9	4166	54.0	0.97
β-blockers	5228	37.3	5134	23.8	<0.0001	2395	31.0	2425	31.4	0.60
Calcium-channel blockers	8907	63.6	9664	44.7	<0.0001	4363	56.5	4395	56.9	0.60
Diuretics	6309	45.1	6486	30.0	<0.0001	2931	38.0	2939	38.1	0.89
Other antihypertensives	2813	20.1	2712	12.5	<0.0001	1295	16.8	1261	16.3	0.46
Antidiabetic drugs										
Oral antidiabetic agents					<0.0001					0.74
0–1	8368	59.8	20593	95.3		6770	87.7	6799	88.1	
2	3500	25.0	821	3.80		756	9.79	728	9.43	
>2	2133	15.2	199	0.92		195	2.53	194	2.51	
Insulin	8722	62.3	6706	31.0	<0.0001	3782	49.0	3777	48.9	0.94
COPD drugs										
Short-actingβ_2_bronchodilators	2856	20.4	2642	12.2	<0.0001	1294	16.8	1298	16.8	0.93
Long-actingβ_2_bronchodilators	200	1.43	187	0.87	<0.0001	82	1.06	92	1.19	0.45
Anticholinergic agent	1960	14.0	2025	9.37	<0.0001	971	12.6	931	12.1	0.33
Inhaled corticosteroids	885	6.32	590	2.73	<0.0001	313	4.05	327	4.24	0.57
systemic corticosteroid	6110	43.6	5884	27.2	<0.0001	2836	36.7	2818	36.5	0.76
Methylxanthine	7818	55.8	8571	39.7	<0.0001	3859	50.0	3858	50.0	0.99
Other drugs										
Statin	6271	44.8	3648	16.9	<0.0001	2375	30.8	2351	30.4	0.68
Aspirin	6531	46.6	6263	29.0	<0.0001	2997	38.8	2981	38.6	0.79

Abbreviation: DCSI, diabetes complications severity index score; ACEI, angiotensin converting enzyme–inhibitor; ARB, angiotensin II receptor–blocker.

For the primary outcome of all-cause mortality, we censored patients at the time of death or until the end of the follow-up, whereas for analyses of metabolic acidosis, we censored patients until the time of events, death, or end of the follow-up on December 31, 2013, whichever came first. We compared the cumulative incidence of mortality over time between metformin users and nonusers through the Kaplan–Meier method and log-rank test. A Cox proportional hazards model was used to compare outcomes while controlling baseline covariates. We performed all analyses according to the initial metformin allocation, irrespective of subsequent changes to other antidiabetic medications. We disclosed results as hazard ratios with 95% confidence intervals (CIs) compared with metformin nonusers. Subgroup analysis with prespecified strata of clinical interest was used to assess effect modification; gender, age (<65 years and ≥65 years), CCI (0, 1, and ≥2), DCSI (0, 1, and ≥2), OADs (0–1, 2, and >2), and insulin (no, yes) were analyzed. We assumed a two-tailed *p* value less than 0.05 as significant. In this study, we used SAS statistical software (Version 9.4 for Windows; SAS Institute, Inc., Cary, NC, USA) for data analysis.

## Results

**Fig 1:**
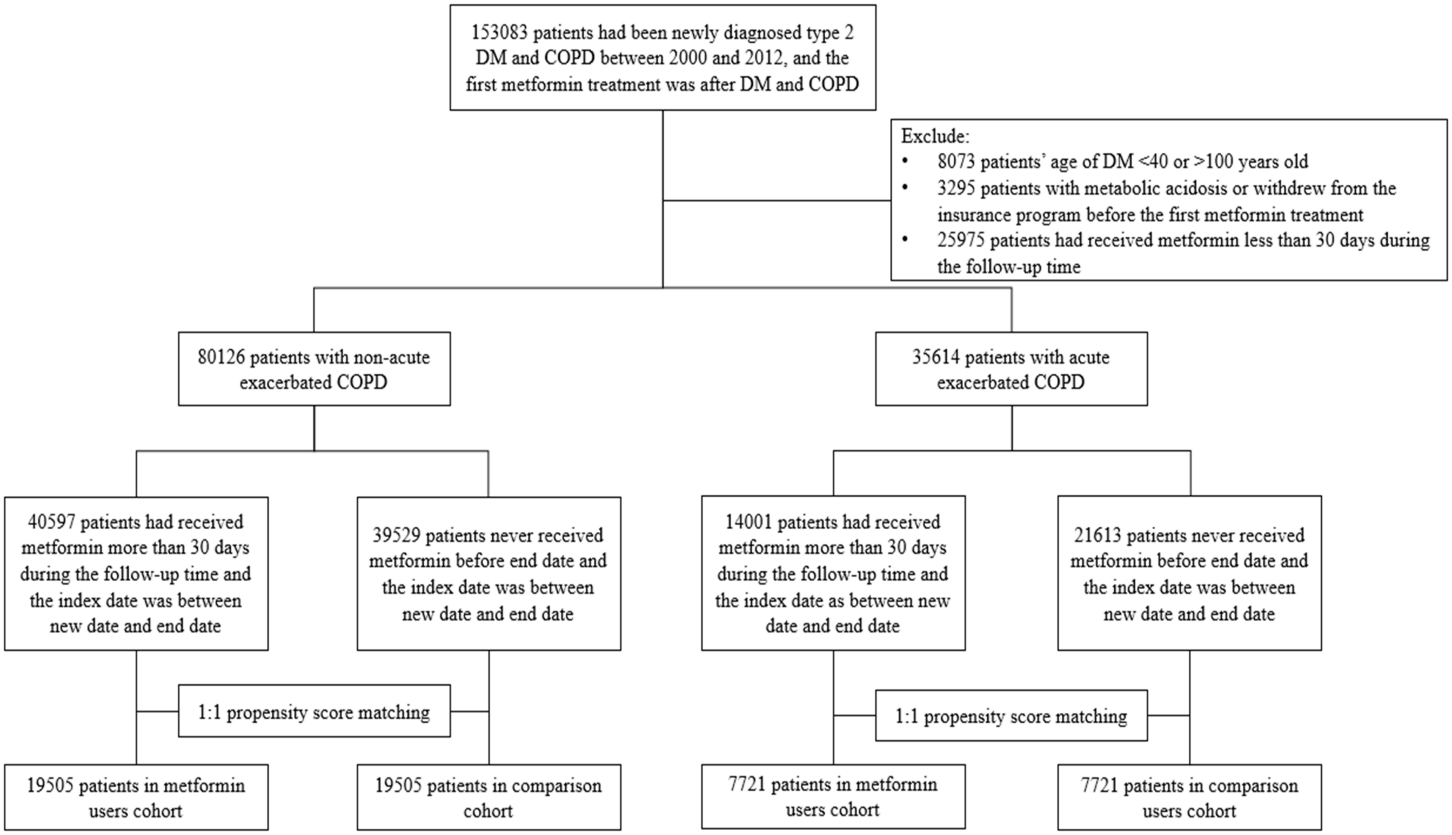
Flow chart of study design and number of patients.

In total, 153,083 patients were newly diagnosed with T2DM and COPD; after excluding ineligible patients, 80,126 patients with T2DM and stable COPD and 35,614 patients with T2DM and exacerbated COPD in the LHDB between January 1, 2000 and December 31, 2012 comprised the overall cohorts of our study (Fig 1).

In total, 40,597 and 39,529 individuals with T2DM and stable COPD were metformin users and nonusers, respectively (Fig 1). After matching participants in a 1:1 ratio according to propensity score, 19,505 and 19,505 patients were included in the outcome analysis as metformin users and nonusers, respectively. The two groups of patients were similar with respect to all covariates (Table 1). The mean age of these two cohorts was 65.4 years. The mean durations of diabetes for metformin users and nonusers were 6.40 years (standard deviation, SD = 3.74) and 6.54 years (SD = 3.71), respectively. The follow-up times for metformin users and nonusers were 3.91 years (SD = 2.63) and 3.92 years (SD = 2.98), respectively.

**Fig 2:**
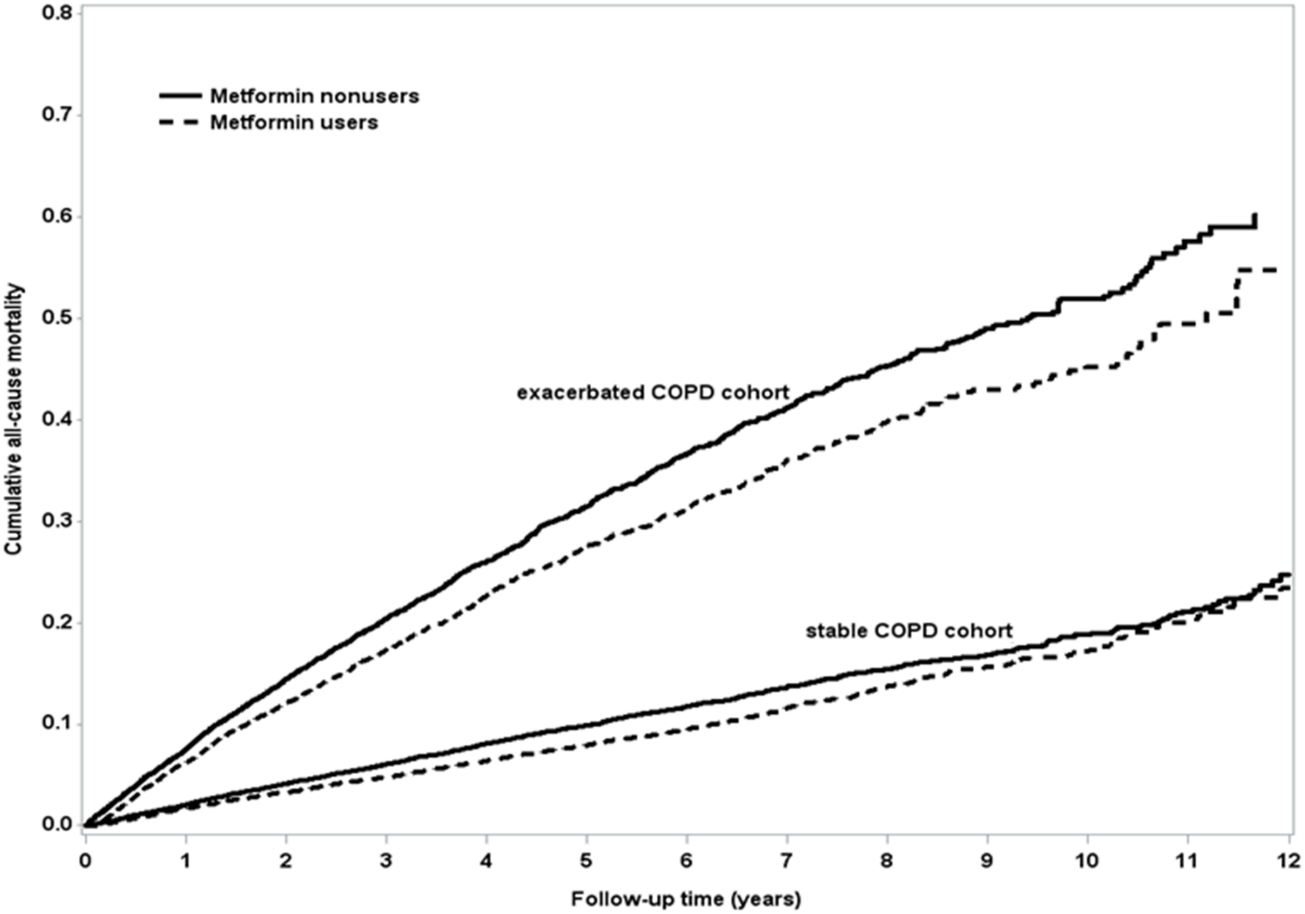
Cumulative all-cause mortality between metformin users and nonusers of patients with T2DM and stable (log-rank test, *p* < 0.0001) or exacerbated COPD (log-rank test, *p* < 0.0001) by Kaplan–Meier curve.

In the matched cohort of patients with T2DM and stable COPD, 1326 (6.80%) of 19,505 metformin users and 1609 (8.25%) of 19,505 nonusers died during follow-up (incidence 174 vs. 211 per 10,000 person–years; crude hazard ratio = 0.83, 95% CI = 0.77–0.89; adjusted hazard ratio [aHR] = 0.84, 95% CI = 0.78–0.91, *p* < 0.0001; Table 3). The difference in survival probability between the metformin users and nonusers was illustrated using a Kaplan–Meier graph (Fig 2), which indicated a higher survival probability for metformin users than for nonusers. The major identifiable causes of death in metformin users included 100 (0.51%) CV deaths (25 ischemic heart disease, 19 sudden cardiac death, 16 HF, 33 stroke, 6 CV hemorrhage, and 1 other CV cause), 1057 (5.42%) non-CV deaths (411 cancers, 101 lung cancers, 85 respiratory disorders, 117 bacterial pneumonia, and 444 others), and 169 (0.87%) undetermined cases. The identifiable causes of death for the metformin nonusers included 135 (0.69%) CV deaths (25 ischemic heart diseases, 22 sudden cardiac death, 20 HF, 58 strokes, 9 CV hemorrhage, and 1 other CV cause), 1262 (6.47%) non-CV deaths (384 cancers, 76 lung cancers, 110 respiratory disorders, 151 bacterial pneumonia, and 617 others); and 212 (1.09%) undetermined cases (Table 4). Compared with nonusers, metformin users had non-significantly lower risk of CV death (aHR = 0.78, 95% CI = 0.59–1.03, *p* = 0.08) and significantly lower risk of non-CV death (aHR = 0.86, 95% CI = 0.79–0.94, *p* = 0.0008, Table 4).

**Table 3:** Incidence and hazard ratios of all-cause mortality of the stable and exacerbated COPD cohorts.

	Metformin non-users			Metformin users			Crude		adjusted	
	Events	Person-	Incidence	Events	Person-	Incidence	Hazard ratio	*p* value	Hazard ratio	*p* value
		year	rate		year	rate	(95% CI)		(95% CI)	
Stable COPD	1609	76402	211	1326	76319	174	0.83(0.77,0.89)	<0.0001	0.84(0.78,0.91)	<0.0001
Exacerbated COPD	1865	24477	762	1567	24661	635	0.83(0.78,0.89)	<0.0001	0.89(0.83,0.96)	0.002

Incidence rate shown per 10,000 person–years. Models adjusted by gender, age, CCI, DCSI, and medications.

**Table 4:** Causes of death of the stable and exacerbated COPD cohorts.

	Stable COPD cohort				Exacerbated COPD cohort			
	Metformin users (n = 19505)	Metformin non-users (n = 19505)	Adjusted		Metformin users (n = 7721)	Metformin non-users (n = 7721)	Adjusted	
	N(%)	N(%)	Hazard ratio (95% CI)	p value	N(%)	N(%)	Hazard ratio (95% CI)	p value
Causes of CV death	100(0.51)	135(0.69)	0.78(0.59,1.03)	0.08	92(1.19)	136(1.76)	0.70(0.52,0.92)	0.01
Ischemic heart disease	25	25			19	29		
Sudden cardiac death	19	22			19	26		
Heart failure	16	20			23	26		
Stroke	33	58			28	43		
Cardiovascular procedure	0	0			0	0		
Cardiovascular hemorrhage	6	9			2	6		
Other cardiovascular causes	1	1			1	6		
Non-cardiovascular causes of death	1057(5.42)	1262(6.47)	0.86(0.79,0.94)	0.0008	1257(16.3)	1380(17.9)	0.97(0.89,1.05)	0.46
Cancer	411	384			306	221		
Lung cancer	101	76			92	64		
Respiratory disorders	85	110			183	200		
Bacterial pneumonia	117	151			234	239		
Others	444	617			534	720		
Undetermined	169(0.87)	212(1.09)			218(2.82)	349(4.52)		

Models adjusted by gender, age, CCI, DCSI, and medications. Codes of ICD-9-CM of diseases or procedures: Ischemic heart disease (MI: 410, 411.0, 412, and 429.79; CAD: 410–414 and 429.2). Sudden cardiac death (sudden cardiac arrest: V12.53, cardiac arrhythmia: 42**7).** HF (398.91, 402.01, 402.11, 402.91, and 428). Stroke (430–438). CV procedures (668.1 and 997.1). CV hemorrhage (aortic aneurysm and dissection: 441; cardiac tamponade: 423.3). Other CV causes (arterial embolism and thrombosis: 444). Cancers (140–208). Lung cancers (162). Respiratory disorders (518.81, 518.82, 518.85, 786.09, 799.1, 96.71, 96.72, 96.04, and 93.9). Bacterial pneumonia (481, 486, 482.41, and 482.8).

**Fig 3:**
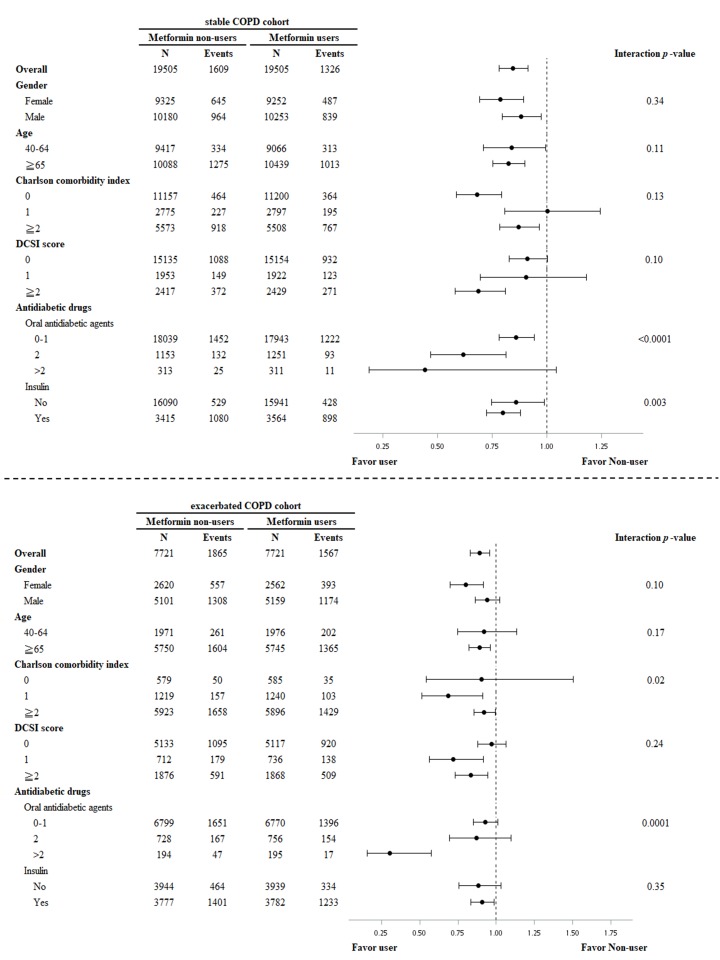
Subgroup analysis of effects of metformin users vs. nonusers on risks of all-cause mortality in patients with T2DM and stable COPD or exacerbated COPD.

Subgroup analysis of T2DM and stable COPD revealed that metformin users, compared with nonusers, had lower risk of all-cause mortality in male and female patients, those <65 and ≥65 years of age, CCI = 0 and ≥2, DCSI = 0 and ≥2, 0–1 and 2 OADs, and with and without insulin therapy. Metformin users who took two OADs or received insulin therapy had prominently lower risk of all-cause mortality (Fig 3).

In total, 14,001 metformin users and 21,613 nonusers were included in the cohort of T2DM with exacerbated COPD (Fig 1). After matching the participants in a 1:1 ratio according to propensity score, 7721 patients each as metformin users and nonusers were included in the outcome analysis. The two groups of patients were similar with respect to all covariates (Table 2). The mean age of two cohorts was 71.8 years. The mean duration of diabetes for metformin users and nonusers was 5.67 years (SD = 3.73) and 5.75 years (SD = 3.56), respectively. The follow-up time of metformin users and nonusers was 3.19 years (SD = 2.41) and 3.17 years (SD = 2.65), respectively. In the matched cohort of T2DM with exacerbated COPD, 1567 (20.30%) of 7721 metformin users and 1865 (24.15%) of 7721 nonusers died during the follow-up (incidence rate 635 vs. 762 per 10,000 patient–years; crude hazard ratio = 0.83, 95% CI = 0.78–0.89; aHR = 0.89, 95% CI = 0.83–0.96; *p* = 0.002; Table 3). The difference in survival probability between the metformin users and nonusers was demonstrated using a Kaplan–Meier graph (Fig 2), which revealed lower cumulative all-cause mortality in metformin users compared with nonusers. The major identifiable causes of death in metformin users were 92 (1.19%) CV deaths (19 ischemic heart diseases, 19 sudden cardiac death, 23 HF, 28 strokes, 2 CV hemorrhage, and 1 other CV cause), 1257 (16.3%) non-CV deaths (306 cancers, 92 lung cancers, 183 respiratory disorders, 234 bacterial pneumonia, and 534 others), and 218 (2.82%) undetermined cases. The identifiable causes of death in metformin nonusers were 136 (1.76%) CV deaths (29 ischemic heart diseases, 26 sudden cardiac death, 26 HF, 43 strokes, 6 CV hemorrhage, and 6 other CV causes), 1380 (17.9%) non-CV deaths (221 cancers, 64 lung cancers, 200 respiratory disorders, 239 bacterial pneumonia, and 720 others), and 349 (4.52%) undetermined cases (Table 4). The metformin users, compared with the nonusers, had significantly lower risk of CV death (aHR = 0.70, 95% CI 0.52–0.92, *p* = 0.01, Table 4).

Subgroup analysis of T2DM and exacerbated COPD revealed that metformin users compared with nonusers had lower risk of all-cause mortality in female patients, those ≥65 years of age, CCI = 1 and ≥2, DCSI of 1 and ≥2, >2 OADs, and with insulin therapy. The metformin users with CCI = 1 or receiving >2 OAD treatments had prominently lower risk of all-cause mortality compared with nonusers (Fig 3).

Exploratory analysis was conducted for the safety outcome of metabolic acidosis. Metabolic acidosis was recorded in 70 (0.36%) of 19,505 metformin users compared with 55 (0.28%) of 19,505 nonusers in the cohort of patients with T2DM and stable COPD. The incidence rates were 9.17 and 7.2 per 10,000 person–years, respectively (crude hazard ratio = 1.28; 95% CI = 0.90–1.83; aHR = 1.34; 95% CI 0.91–1.97; *p =* 0.13; models were adjusted by sex, age, CCI, DCSI, and medications as listed in Table 1), and no statistical significance was noted. Metabolic acidosis was recorded in 106 (1.37%) of 7721 metformin users compared with 94 (1.22%) of 7721 nonusers in the cohort of patients with T2DM and exacerbated COPD. The incidence rates were 43 and 38.4 per 10,000 person–years, respectively (crude hazard ratio = 1.12; 95% CI = 0.84–1.47; aHR = 1.21, 95% CI = 0.89–1.63; *p =* 0.22, models were adjusted by sex, age, CCI, DCSI, and medications as listed in Table 2), and no statistical significance was noted.

## Discussion

To our knowledge, this is the largest study comparing the long-term effects of metformin use and nonuse in patients with T2DM and COPD. We used 1:1 propensity score matching to compare all-cause mortality and metabolic acidosis between metformin users and nonusers in cohorts of 39,010 and 15,442 patients with T2DM and stable or exacerbated COPD, respectively. The results revealed that metformin use could significantly decrease the risk of all-cause mortality in patients with T2DM and stable and exacerbated COPD. This protective effect was consistent over subgroup analysis of gender, age, comorbidity, DCSI, and other antidiabetic drug use. In the cohort of patients with T2DM and stable COPD, the metformin users had significantly lower risk of non-CV death compared with the nonusers, whereas in the cohort of T2DM patients with exacerbated COPD, metformin users had significantly lower risk of CV death compared with the nonusers.

CH Tseng conducted a population-based study and demonstrated preventive effects of metformin against the development of COPD in patients with T2DM [29]. Hitchings et al. conducted a retrospective observational study of 144 patients with combined T2DM and COPD from clinical coding data. Out of 144, 51 were metformin users, and 79 were metformin nonusers; the metformin group was associated with a survival benefit (log-rank test, *p* = 0.011) [18]. Hitchings et al. also directed a randomized controlled trial to evaluate the anti-hyperglycemia, anti-inflammation, and clinical outcomes of metformin use on patients with exacerbated COPD but without diabetes. A total of 52 participants were randomized (34 to metformin, 18 to placebo), and no significant between-group difference was observed in concentration of C-reactive protein or clinical outcomes [19]. Our study was consistent with Hitchings’s but with a large population-based and nationwide study. After 1:1 propensity score matching, metformin users displayed lower risk of all-cause mortality in patients with both stable and exacerbated COPD compared with nonusers.

The decreased mortality of metformin-using patients with T2DM and COPD might be caused by the following plausible mechanisms: 1. Metformin mainly activates AMPK to up-regulate glucose transporter 4 genes to increase glucose uptake to decrease hyperglycemia and oxidative stress. 2. Metformin could exhibit anti-inflammatory effects, reduce airway inflammation [13], and decrease epithelial permeability to reduce bacterial growth in the airway [30]. 3. Metformin could induce adiponectin, which might stimulate AMPK and prevent lipid accumulation by increasing β-oxidation of free fatty acids [31]. 4. AMPK is an essential mediator of tumor-suppressor liver kinase B1 (LKB1); through inducing LBK1, metformin could have anticancer and anti-aging effects [32]. 5. The activation of AMPK could reprogram cellular metabolism and enforce metabolic checkpoints by acting on mammalian targets of rapamycin complex 1 and other molecules for regulating cell growth and metabolism [33].

The subgroup analysis of T2DM and stable COPD revealed that metformin users, compared with the nonusers, who took two OADs or received insulin therapy had prominently lower risk of all-cause mortality, suggesting that in metformin users with stable COPD, intensive glycemic control had survival benefits. The subgroup analysis of T2DM and exacerbated COPD revealed that metformin users receiving >2 OAD treatments had lower risk of all-cause mortality, but insulin therapy didn’t have such benefit. This might indicate that patients with T2DM and exacerbated COPD were more vulnerable, too intensive insulin therapy might induce higher risk and had no survival benefits.

Our study also indicates that metformin use has the tendency to lower the risk of CV death in patients with T2DM and stable COPD, and it significantly lowers the risk of CV death in patients with T2DM and exacerbated COPD. In the category of CV death, metformin seems to decrease more death of stroke. Metformin could lower the risk of non-CV death in patients with T2DM and stable COPD, but it could not lower the risk of non-CV death in patients with T2DM and exacerbated COPD. Patients with exacerbated COPD had deteriorating lung function with shorter survival; thus, it might be too late to start metformin doses in these patients. In the category of non-CV death, metformin decreases respiratory death and death by bacterial pneumonia but increases the risk of death by cancer; however, metformin was reported to decrease the rate of mortality from several cancers [34]. We need to conduct stringent studies to observe the CV and respiratory outcomes of metformin use in patients with T2DM and COPD.

COPD is a chronic inflammatory lung disease with slow progression, and most patients with COPD are elderly. Therefore, COPD is a lung-aging disease that is accelerated by exogenous oxidative stress [35]. Recently, some anti-aging molecules (such as metformin) were identified that might open up new avenues for COPD treatment [36]. The effect and safety of those molecules in the lungs have not been fully evaluated. Our study demonstrates that metformin use in patients with COPD could lower the risk of all-cause mortality, which might provide a clue that metformin use in patients with COPD could prevent premature lung aging and prolong survival compared with metformin nonuse.

Interfering with the respiratory oxidation in mitochondria, metformin could suppress gluconeogenesis from several substrates, such as lactate, pyruvate, glycerol, and amino acid. When patients were in hypoxic conditions (sepsis, congestive HF, and hypoxic respiratory condition), metformin increased the risk of lactic acidosis [37]. The US Food and Drug Administration [38] and British National Formulary [39] have recommended that metformin be withheld in conditions associated with hypoxemia. This advice restricted the clinical use of metformin among patients with T2DM and COPD. Despite the widespread use of metformin, only rare cases of patients with COPD developing lactic acidosis were reported [40]. Our study demonstrated that metformin use for stable and exacerbated COPD did not increase the risk of metabolic acidosis.

Our study has some advantages. First, our study is a population-based, real-world finding, with large series and long-term follow-up data that were collected from the national insurance database. Second, both the control groups were well matched by propensity scores for sex, age, CCI, DCSI, other antidiabetic agents, antihypertensive drugs, COPD drugs, statin, and aspirin to decrease probable confounding factors. However, the influence of residual confounding from an imbalance of unavoidable baseline covariates cannot be ruled out.

This study also has several limitations. First, the NHIRD cannot provide information of patients’ family history, body mass index, alcohol consumption, cigarette smoking, or physical activity, all of which might influence mortality. Second, the NHIRD database lacks pulmonary function tests and records of patients’ symptoms and signs; therefore, we could not calculate the COPD severity scores. We used clinical pictures to separate patients with stable and exacerbated COPD and observed whether metformin use yielded different outcomes between disease severities. This database lacks data on biochemical blood test results, such as hemoglobin A1C, blood glucose level, lipid profiles, and renal function; therefore, we could not use the data to evaluate the severity of diabetes. Instead, we used DCSI to balance the diabetes severity between metformin users and nonusers. Third, because we could not link data to the national death registry, our all-cause mortality was an estimated mortality rate. We used the last primary diagnosis of death to determine CV and non-CV causes of death in patients with T2DM and COPD who used metformin versus those who did not. The accuracy of diagnoses based on the ICD-9-CM codes in this database might affect the study findings. However, the NHI regularly censors the charts and evaluated the accuracy of claims files. Erroneous disease coding would receive no reimbursement and accordingly result in fines. The high accuracy of data in the NHIRD has been proven by several studies [41, 42].

In conclusion, our study disclosed that metformin use in patients with T2DM and COPD was beneficial in terms of survival compared with metformin nonuse; no significant difference in the risk of metabolic acidosis was noted between stable and exacerbated COPD. However, additional studies are warranted to establish the optimal application of metformin in real-world practice.

## Conclusions

In this large nationwide, population-based cohort study, metformin use in patients with T2DM and COPD could lower the risk of all-cause mortality without increasing the risk of metabolic acidosis.

## References

[1] Rabe KF, Watz H. Chronic obstructive pulmonary disease. Lancet.2017;389(10082):1931–1940. 10.1016/S0140-6736(17)31222-9 28513453

[2] Buist AS, McBurnie MA, Vollmer WM, Gillespie S, Burney P, Mannino DM, et al International variation in the prevalence of COPD (the BOLD Study): a population-based prevalence study. Lancet.2007;370:741–750. 10.1016/S0140-6736(07)61377-4 17765523

[3] Adeloye D, Chua S, Lee C, Basquill C, Papana A, Theodoratou E, et al Global and regional estimates of COPD prevalence: systematic review and meta-analysis.J Glob Health. 2015;5(2):020415. 26755942 10.7189/jogh.05-020415 PMC4693508

[4] GBD 2015 Mortality and Causes of Death Collaborators. Global, regional, and national life expectancy, all-cause mortality, and cause-specific mortality for 249 causes of death, 1980–2015: a systematic analysis for the Global Burden of Disease Study 2015. Lancet.2016;388:1459–1544. 10.1016/S0140-6736(16)31012-1 27733281 PMC5388903

[5] Rana JS, Mittleman MA, Sheikh J, Hu FB, Manson JE, Colditz GA, et al Chronic obstructive pulmonary disease, asthma, and risk of type 2 diabetes in women. Diabetes Care. 2004;27:2478–2484. 15451919 10.2337/diacare.27.10.2478

[6] Niewoehner DE, Erbland ML, Deupree RH, Collins D, Gross NJ, Light RW, et al Effect of systemic glucocorticoids on exacerbations of chronic obstructive pulmonary disease. Department of Veterans Affairs Cooperative Study Group. N Engl J Med. 1999;340:1941–1947. 10.1056/NEJM199906243402502 10379017

[7] Chatila WM, Thomashow BM, Minai OA, Criner GJ, Make BJ. Comorbidities in chronic obstructive pulmonary disease. Proc Am Thorac Soc. 2008;5:549–555. 10.1513/pats.200709-148ET 18453370 PMC2645334

[8] International Diabetes Federation. IDF diabetes atlas. 7th ed Brussels: International Diabetes Federation; 2015.

[9] GBD 2015 Disease and Injury Incidence and Prevalence Collaborators. GBD 2015 Disease and Injury Incidence and Prevalence Collaborators. Global, regional, and national incidence, prevalence, and years lived with disability for 310 diseases and injuries, 1990–2015: a systematic analysis for the Global Burden of Disease Study 2015. Lancet. 2016;388:1545–1602. 27733282 10.1016/S0140-6736(16)31678-6 PMC5055577

[10] Caughey GE, Roughead EE, Vitry AI, McDermott RA, Shakib S, Gilbert AL. Comorbidity in the elderly with diabetes: identification of areas of potential treatment conflicts. Diabetes Res Clin Pract. 2010;87:385–393. 10.1016/j.diabres.2009.10.019 19923032

[11] Gläser S, Krüger S, Merkel M, Bramlage P, Herth FJ. Chronic obstructive pulmonary disease and diabetes mellitus: a systematic review of the literature. Respiration.2015;89(3):253–264. 10.1159/000369863 25677307

[12] Stumvoll M, Nurjhan N, Perriello G, Dailey G, Gerich JE. Metabolic effects of metformin in non-insulin-dependent diabetes mellitus. N Engl J Med. 1995;333:550–554. 10.1056/NEJM199508313330903 7623903

[13] Park CS, Bang BR, Kwon HS, Moon KA, Kim TB, Lee KY, et al Metformin reduces airway inflammation and remodeling via activation of AMP-activated protein kinase. Biochem Pharmacol.2012;84:1660–1670. 10.1016/j.bcp.2012.09.025 23041647

[14] Zhou G, Myers R, Li Y, Chen Y, Shen X, Fenyk-Melody J, et al Role of AMP-activated protein kinase in mechanism of metformin action. J Clin Invest. 2001;108:1167–1174. 10.1172/JCI13505 11602624 PMC209533

[15] Song P, Zou MH. Regulation of NAD(P)H oxidases by AMPK in cardiovascular systems. Free RadicBiol Med. 2012;52:1607–1619. 10.1016/j.freeradbiomed.2012.01.025 PMC3341493 22357101

[16] Sexton P, Metcalf P, Kolbe J. Respiratory effects of insulin sensitization with metformin: a prospective observational study. COPD. 2014;11:133–142. 10.3109/15412555.2013.808614 23848509

[17] Kim HJ, Lee JY, Jung HS, Kim DK, Lee SM, Yim JJ, et al The impact of insulin sensitisers on lung function in patients with chronic obstructive pulmonary disease and diabetes. Int J Tuberc Lung Dis. 2010;14(3):362–367. 20132629

[18] Hitchings AW, Archer JR, Srivastava SA, Baker EH. Safety of metformin in patients with chronic obstructive pulmonary disease and type 2 diabetes mellitus.COPD.2015;12(2):126–131. 10.3109/15412555.2015.898052 25938184

[19] Hitchings AW, Lai D, Jones PW, Baker EH. Metformin in severe exacerbations of chronic obstructive pulmonary disease: a randomised controlled trial. Thorax. 2016;71:587–593. ISSN 0040-6376 10.1136/thoraxjnl-2015-208035 26917577 PMC4941151

[20] Cheng TM. Taiwan’s new national health insurance program: genesis and experience so far. Health Aff (Millwood). 2003;22:61–76. 12757273 10.1377/hlthaff.22.3.61

[21] Burge S, Wedzicha JA. COPD exacerbations: definitions and classifications. Eur Respir J Suppl.2003;41:46s–53s. 12795331 10.1183/09031936.03.00078002

[22] Charlson ME, Pompei P, Ales KL, MacKenzie CR. A new method of classifying prognostic comorbidity in longitudinal studies: development and validation. J Chronic Dis. 1987;40:373–383. 3558716 10.1016/0021-9681(87)90171-8

[23] Young BA, Lin E, Von Korff M, Simon G, Ciechanowski P, Ludman EJ, et al Diabetes complications severity index and risk of mortality, hospitalization, and healthcare utilization. Am J Manag Care.2008;14:15–23. 18197741 PMC3810070

[24] Wu CY, Chen YJ, Ho HJ, Hsu YC, Kuo KN, Wu MS, et al Association between nucleoside analogues and risk of hepatitis B virus–related hepatocellular carcinoma recurrence following liver resection. JAMA.2012;308(18):1906–1914. 23162861 10.1001/2012.jama.11975

[25] Hicks KA, JamesHung HM, Mahaffey KW, Mehran R, Nissen SE, Stockbridge NL, et al; on behalf of the Standardized Data Collection for Cardiovascular Trials Initiative. Standardized definitions for cardiovascular and stroke end point events in clinical trials. Draft Definitions for CDISC [cited 2014 July 3]. https://mdepinet.org/wp-content/uploads/S_1_6_Hicks.pdf

[26] D’Agostino RB Jr. Propensity score methods for bias reduction in the comparison of a treatment to a non-randomized control group. Stat Med.1998;17:2265–2281. 9802183 10.1002/(sici)1097-0258(19981015)17:19<2265::aid-sim918>3.0.co;2-b

[27] Glynn RJ, Schneeweiss S, Sturmer T. Indications for propensity scores and review of their use in pharmacoepidemiology. Basic Clin Pharmacol Toxicol.2006;98:253–259. 10.1111/j.1742-7843.2006.pto_293.x 16611199 PMC1790968

[28] Iezzoni LI. Risk adjustment for measuring healthcare outcomes. 2nd ed Chicago: Health Administration Press; 1997.

[29] Tseng CH. Metformin and risk of chronic obstructive pulmonary disease in diabetes patients. Diabetes Metab. 2018 5 17 10.1016/j.diabet.2018.05.001 [Epub ahead of print] 29804817

[30] Garnett JP, Baker EH, Naik S, Lindsay JA, Knight GM, Gill S, et al Metformin reduces airway glucose permeability and hyperglycaemia-induced Staphylococcus aureus load independently of effects on blood glucose.Thorax.2013;68(9):835–845. 10.1136/thoraxjnl-2012-203178 23709760 PMC3756442

[31] Rena G, Hardie DG, Pearson ER. The mechanisms of action of metformin. Diabetologia. 2017 60(9):1577–1585. 10.1007/s00125-017-4342-z 28776086 PMC5552828

[32] Dowling RJ, Zakikhani M, Fantus IG, Pollak M, Sonenberg N. Metformin inhibits mammalian target of rapamycin-dependent translation initiation in breast cancer cells. Cancer Res.2007;67:10804–10812. 10.1158/0008-5472.CAN-07-2310 18006825

[33] Luo Z, Zang M, Guo W. AMPK as a metabolic tumor suppressor: control of metabolism and cell growth. Future Oncol.2010;6:457–470. 10.2217/fon.09.174 20222801 PMC2854547

[34] Libby G, Donnelly LA, Donnan PT, Alessi DR, Morris AD, Evans JM. New users of metformin are at low risk of incident cancer: a cohort study among people with type 2 diabetes. Diabetes Care. 2009;32:1620–1625. 10.2337/dc08-2175 19564453 PMC2732153

[35] Lowery EM, Brubaker AL, Kuhlmann E, Kovacs EJ. The aging lung. Clin Interv Aging.2013;8:1489–1496. 10.2147/CIA.S51152 24235821 PMC3825547

[36] Ito K, Mercado N. STOP accelerating lung aging for the treatment of COPD. Exp Gerontol.2014;59:21–27. 10.1016/j.exger.2014.03.014 24709339

[37] Kirpichnikov D, McFarlane SI, Sowers JR. Metformin: an update. Ann Intern Med.2002;137(1):25–33. 12093242 10.7326/0003-4819-137-1-200207020-00009

[38] Bristol-Myers Squibb Company. Glucophage (metformin hydrochloride) tablets. Package Insert. Princeton, NJ, USA; 2009. http://packageinserts.bms.com/pi/pi_glucophage.pdf.

[39] British National Formulary (online). London: BMJ Group and Pharmaceutical Press; 2013 http://www.bnf.org/bnf/index.htm.

[40] Abbasi AA, Kasmikha R, Sotingeanu DG. Metformin-induced lactic acidemia in patients with type 2 diabetes mellitus. Endocr Pract. 2000;6:442–446. 10.4158/EP.6.6.442 11155215

[41] Cheng CL, Kao YH, Lin SJ, Lee CH, Lai ML. Validation of theNational Health Insurance Research Database with ischemic stroke cases in Taiwan. Pharmacoepidemiol Drug Saf.2011;20:236–242. 10.1002/pds.2087 21351304

[42] Yu YB, Gau JP, Liu CY, Yang MH, Chiang SC, Hsu HC, et al. A nation-wide analysis of venous thromboembolism in 497,180 cancer patients with the development and validation of a risk-stratification scoring system. Thromb Haemost.2012;108:225–235. 10.1160/TH12-01-0010 22534880

